# Adaptive health technology assessment to facilitate priority setting in low- and middle-income countries

**DOI:** 10.1136/bmjgh-2020-004549

**Published:** 2021-04-26

**Authors:** Cassandra Nemzoff, Francis Ruiz, Kalipso Chalkidou, Abha Mehndiratta, Lorna Guinness, Francoise Cluzeau, Hiral Shah

**Affiliations:** 1 International Decision Support Initiative, Center for Global Development, London, UK; 2 Global Health and Development Group, Imperial College London Department of Infectious Disease Epidemiology, London, UK; 3 London School of Hygiene and Tropical Medicine, London, UK; 4 MRC Center for Global Infectious Disease Analysis, Imperial College London Department of Infectious Disease Epidemiology, London, UK

**Keywords:** health economics, health policy, health systems, public health

**Summary box**

- There is a growing appetite for health technology assessment (HTA) in low-and middle-income countries (LMICs) to better inform healthcare priority setting.
- However, LMICs are sometimes constrained by limited capacity, data, time and priority setting governance structures to carry out HTA.
- LMICs may benefit from adaptive HTA (aHTA), which we define as a broad term for HTA methods and processes which are fit-for-purpose and focus on context-specific practicality constraints.
- aHTA can leverage or adapt available international data, economic evaluations, models and/or decisions from the published literature or established HTA agencies to inform policy decisions, while accounting for uncertainty considerations.
- aHTA should be pragmatic, though still informed by key HTA principles such as transparency, independence, consultation and contestability.
- More work is needed to design, support and test bespoke aHTA for LMICs to better understand its strengths and limitations.

## Introduction

Traditional health technology assessment (HTA) is a policy-based research process, which aims to improve the efficiency and equity of the healthcare system with the limited financial resources available in healthcare.^1^ In various countries, traditional HTA has been ‘institutionalised’—through the development of dedicated agencies with accepted norms and rules that guide explicit priority setting—over years or decades. These agencies use time-consuming, data intensive and systematic methods and processes which require health economics expertise and resources to make recommendations on how to allocate finite resources.^2^

There is a growing appetite for HTA and its eventual institutionalisation in low- and-middle income countries (LMICs) driven in part by WHO’s recommendation for it to be a critical component to achieving universal health coverage.^3^ While there are notable LMIC exceptions of introducing and institutionalising HTA (eg, Thailand, Colombia, Brazil and India), others may be constrained by limited technical and administrative capacity, paucity of data, time and governance structures to carry out HTA.^4^

A more pragmatic approach which we define in this paper as ‘adaptive HTA’ (aHTA) is one which uses various expedited or flexible methods and processes that are ‘fit for purpose’ and could help to tackle some of these challenges faced by LMICs. Here, we suggest how policy makers, researchers, clinicians and donors can collaborate and support the development and uptake of aHTA for LMICs to enable expedited evidence-based decision making in these countries as one part of the journey towards HTA institutionalisation.

## What is adaptive health technology assessment?

Ideally, all health policy decisions should involve the use of HTA processes and methods which offer quality, context-specific and locally relevant evidence. However, where the ‘gold standard’ of HTA is not immediately possible, countries may seek to expedite or adapt some aspects of the HTA approach.^5^ The broad concept of aHTAs is not new; it is used in high-income countries such as the UK, Canada and the European Union.^6 7^ These approaches are largely focused on expedited processes to respond to time constraints, for example, in the case of a new technology or a public health emergency.^8^ However, there are limited examples from LMICs transferring the same types of approaches, as LMICs are more likely to be constrained not only by time, but also by capacity, resources and data. Hence there is a need for HTA processes, methods and analytics that can be adapted to suit LMIC HTA constraints.

We seek to define aHTA as a blanket approach for HTA methods and processes which are fit-for-purpose and focused on context-specific practicality considerations. Methodologically, aHTA may leverage or adapt available international data, economic evaluations, models and/or decisions from the published literature or established HTA agencies to expedite policy decisions while adequately accounting for concerns of transferability and uncertainty.^9^ The aHTA process should be pragmatic, though still informed by key HTA principles such as transparency, independence, consultation and contestability.

There is no ‘one-size-fits-all’ approach to aHTA as no two health systems are alike, but generally components of traditional HTA can be modified or adapted pragmatically to suit LMICs’ needs (table 1). Hence, aHTA may benefit LMICs which either have a nascent HTA agency or not one at all, and do not receive HTA submissions from pharmaceutical companies. In circumstances where an LMIC does have an active HTA agency with adequate capacity to appraise evidence, it may be feasible to receive pharmaceutical dossiers as part of the HTA process.

**Table 1:** Methods and trade-offs for adapting traditional health technology assessment (HTA) in low- and middle-income countries (LMICs)

	Traditional HTA	Adaptive HTA in LMICs*	Trade-offs
Timeline	8–12 months+	1–6 months	Level of comprehensiveness. Speed.
Topic selection	Detailed topic selection process with established criteria and fits government priorities.	No process or Opportunistic process or Minimal criteria.	Identifies low-hanging fruits. Local relevance. Range of topics.
Analysis	De novo economic evaluation (eg, cost-effectiveness analysis).	Price benchmarking or Literature reviews or Adapted economic evaluation or Outsourced economic evaluation.	Accuracy. Quality. (Un)certainty. Builds capacity. Leverages available data.
Data sourcing	Local studies+primary data collection and systematic literature review/meta analyses as needed.	Pragmatic/sources known to authors.	Level of comprehensiveness.
Appraisal	Multistakeholder group guided by agreed principles appraises evidence and makes policy recommendations.	No appraisal or Modified appraisal process.	(Sub)optimal decisions. Level of HTA system improvement and health system strengthening.
Implementation	Wide ranging policy changes could include adjustment to health benefits packages, essential medicines lists (including appropriate indications), price negotiations, reimbursement decisions, clinical guidelines, care pathways and quality standards.*		(Sub)optimal allocation of resources. Mobilises HTA institutionalisation.

Table 1 demonstrates potential different approaches for each step of a traditional HTA versus an adapted HTA for the LMIC context. Depending on the adaptation(s) selected, a range of potential trade-offs could be associated with each of these steps which should be considered when using aHTA, as well as the alternative of using no evidence at all.

*While aHTA and traditional HTA can inform similar policy decisions, aHTAs cannot be used for all technologies, as discussed below.

aHTA, adaptive HTA.

## Adaptive health technology assessment methods

There are numerous aHTA methods which vary by scope, approach, complexity and mix of data sources used depending on contexts-specific constraints.^10^ The loosely categorised examples below demonstrate the breadth of these approaches which we are aware of in LMICs.

### Expedited process

The Filipino HTA guidelines set out a rapid review process for public health emergencies.^11^

### Adaptation of international data sets

The World Bank’s Health Interventions Prioritisation Tool and Disease Control Priorities have consolidated international evidence on burden of disease, cost and cost-effectiveness, which have been adapted to inform benefits package design in Afghanistan, Armenia, Cot d’Ivoire, Eswatini, Pakistan and Zimbabwe.^12 13^

### Literature reviews and synthesis

In Vietnam and Romania, aHTAs gathered and synthesised international evidence on safety, clinical efficacy/effectiveness, cost-effectiveness and clinical guidelines for high-cost drugs. Potential savings from rational use of medicines were then calculated.^14 15^

### Price benchmarking

Also in Romania and Serbia ‘indirect’ cost-effectiveness analyses compared a set of high-cost drugs to a gross domestic product-adjusted list price in benchmark countries (eg, UK, USA, Australia) to ascertain the maximum value at which each medicine might be cost-effective locally.^15 16^

### Modelling

In South Africa, a UK model was adapted to evaluate early breast cancer treatment using the Mullins checklist—a model adaptation tool.^17^ Similar model adaptations were done in Tunisia and Jordan.^18 19^

Notably, these are analyses which have been carried out as one part of the HTA process, though the extent to which they have influenced policy or been implemented may be varied or absent (eg, Romania).^9^

## Limitations of using adaptive health technology assessments

The primary incentive for using aHTA in LMICs is to serve policy makers who may be short on capacity, resources, time or bandwidth to take decisions for various government processes such as budget negotiations, ministerial and parliamentary meetings, procurement contracting and tariff negotiations.^20^ In such situations, pursuing an aHTA approach represents a practical and useful option; rather than taking up to 1–2 years for a full HTA process from topic selection to implementation as is perhaps usual under more traditionally applied HTA frameworks, aHTA provides relevant evidence quickly and reduces the domestic analytical burden.

However, aHTA also has its limitations, the most important of which is transferability of international data and information to the local setting, a process which can increase the uncertainty of results. Even for topics which are well studied in other countries, the evidence is often from high- or middle-income countries that have different health systems, healthcare costs, patient characteristics, burdens of disease, value judgements and provider/clinical practice norms than the country under study.^21^ This is further complicated if the evidence used comes from countries where the HTA recommendations are linked with academic and commercial in confidence data (such as the UK), as well as by the publication bias towards technologies with positive cost-effectiveness results. If not adequately acknowledged in the appraisal process using available approaches for assessing transferability with the right expertise in health economics, reliance on misinterpreted international evidence risks leading to suboptimal decision making.^9 22^ Furthermore, if evidence for many technologies is quickly reviewed without such expertise, it could result in a disproportionate number of cost-ineffective technologies being covered, putting unnecessary pressure on the sustainability of public finances as it did in Romania using a rapid ‘scorecard approach’.^23^

In addition to transferability, aHTAs can generally only be conducted on topics for which international data and/or models are available. For topics which are not well-studied globally, de novo collection of local data is required. Depending on the topic and availability of locally relevant data, aHTA may also demand substantial local clinical expertise to understand local health system constraints, clinical pathways and outcomes, all of which are critical to transferring evidence from other jurisdictions.

Furthermore, aHTA may not build the needed wide-ranging capacity—in epidemiological and medical statistics, health economics, HTA processes, evidence appraisal and translation of economic evidence to policy—or create the incentives for building capacity to support HTA institutionalisation.^9^ If countries rely solely on aHTA as an approach to priority setting despite its limitations, sustainable HTA infrastructure and processes may never be built and reliance on development partners may continue.

Finally, while the HTA process has been undertaken in many LMICs and adapted in various ways, publications which detail HTA modifications, benefits, pitfalls and lessons learnt are limited. Moreover, we are not aware of any publication which assesses an aHTA approach against a more traditional one to empirically understand these benefits and pitfalls.

## Benefits of using adaptive health technology assessment as a ‘tool’ in the toolbox

The limitations of aHTA make it clear that while it can offer some relevant and adequately nuanced evidence, which is better than no evidence at all, it is not a replacement for traditional HTA approaches, even those based on more expedited processes but still requiring significant expertise (eg, UK National Institute for Health and Care Excellence (NICE) Single Technology Appraisal). Rather, it is possible that aHTA, if fit-for-purpose, could be a permanent tool in a larger HTA toolbox as it already is in many countries, and support the long-term uptake of HTA. We have found through our work at the international Decision Support Initiative that doing HTA rather than *talking* about HTA is a useful and impactful means of sensitising key stakeholders on the processes and analytics required for good HTA. Through lived experiences, aHTA can spark demand for future HTAs and uncover key data gaps that need to be addressed for use in HTA.

Furthermore, aHTA saves time by identifying ‘low-hanging fruits,’—well-studied technologies which are known to be cost saving, highly cost-effective or very cost-ineffective internationally—minimising the amount of effort required for review. This can allow resource space to be made available for the conduct of more intensive HTAs dedicated to local priorities and technologies that are not well studied in other countries or those that are well studied but there is greater uncertainty about their marginal benefits and costs. Local capacity building can then be a much greater feature in the conduct of such HTAs.

For the future, aHTA requires developing processes, governance structures and analytics that can be leveraged to support a fully institutionalised HTA model. In multiple countries, aHTA has created the impetus for small HTA ‘core teams’, mini topic selection processes, and appraisal processes which can be directly built on for HTA institutionalisation. Policy-makers are also keen to incorporate local data into the aHTA, including for example, cost, epidemiology and coverage rates, which in turn demands locally relevant, more complex, analysis.^24^ In the first instance, there are a few initial steps that researchers, policy makers, and donors could take to support the uptake of aHTA:

- For HTA practitioners, write and publish peer-reviewed examples of aHTAs for global knowledge sharing, detailing where HTAs were adaptive, how they were modified and strengths, weaknesses and lessons learnt.
- For researchers, develop, test and validate a set of standardised approaches for conducting aHTAs in LMICs—drawing on lessons learnt from other LMICs’ HTA journeys—articulating strengths, weaknesses and uncertainty of each approach and identifying necessary skill sets for implementation.
- For policy makers, leverage aHTA as one mechanism for evidence-based decision making, identify priority topics for aHTA and those which demand more detailed analysis, build processes and governance structures which include aHTA, or even develop a ‘reference case’ for the conduct of aHTA.
- For clinicians, develop clinical practice guidelines, pathways and health benefit packages that are more cost conscious and informed by aHTA approaches at least initially, which are updated subsequently if evidence from a traditional HTA becomes available.
- For donors, support the uptake of aHTA and long-term HTA institutionalisation through making key investments such as capacity building, model sharing, model databases, and Incremental Cost-Effectiveness Ratio (ICER)databases (eg, Tufts Cost-Effectiveness Analysis Registry), as well as including HTA uptake as a key indicator for sustainability and aid transition.

## Conclusion

Policy makers in many LMICs are often having to make health financing decisions for their available resources with limited information. Despite limitations, aHTA frameworks can offer evidence where there may otherwise be none while demonstrating the uses of HTA, uncovering gaps in data and capacity, and facilitating the use of HTA going forward.

## Data Availability

Data sharing not applicable as no datasets generated and/or analysed for this study.
